# Supplementary material for: A Likelihood Approach for Real-Time Calibration of Stochastic Compartmental Epidemic Models
Source: PLoS Comput Biol. 2017 Jan 17;13(1):e1005257. doi: 10.1371/journal.pcbi.1005257 (PMC5240920; doi:10.1371/journal.pcbi.1005257)
Supplement: S1 File — (TAR.GZ) [file pcbi.1005257.s014.tar.gz › HSPH_Online-SI-Revision/IPoi09/n100-severe/n100-severe_BENCH2_table0.pdf]

1	newly 3 weeks spec	5129.	7079.75	{5121., 9199.}	41.4039	0.145601	newly 3 weeks cumul	17951.	23670.4	{18680., 28398.}	35.4106	0.133784	newly 5 weeks spec	3152.	5093.05	{3294., 7473.}	63.5662	0.194385	newly 5 weeks cumul	25181.	34838.3	{26267., 43394.}	40.6398	0.143835	newly 50 weeks cumul	33334.	54179.3	{37690., 73214.}	62.8896	0.197296
2	newly 3 weeks spec	3271.	8186.66	{5045., 13054.}	150.28	0.373489	newly 3 weeks cumul	16291.	31890.5	{23201., 39298.}	95.7554	0.282662	newly 5 weeks spec	952.	4084.15	{2273., 6653.}	329.007	0.580379	newly 5 weeks cumul	19055.	41887.	{28826., 59401.}	119.822	0.328298	newly 57 weeks cumul	20150.	49710.3	{32755., 73141.}	146.701	0.373195
3	newly 3 weeks spec	3370.	6358.2	{3607., 11159.}	90.5171	0.238695	newly 3 weeks cumul	19722.	28662.8	{18780., 45181.}	49.3016	0.158569	newly 5 weeks spec	834.	2511.43	{1273., 4569.}	201.13	0.416342	newly 5 weeks cumul	22331.	35232.	{22301., 56843.}	60.7612	0.184141	newly 58 weeks cumul	23238.	39033.	{24193., 63798.}	70.2086	0.204547
4	newly 3 weeks spec	5397.	6374.69	{3834., 11325.}	36.9222	0.131984	newly 3 weeks cumul	21357.	25256.	{17153., 39063.}	28.9189	0.107445	newly 5 weeks spec	2735.	3253.58	{1806., 5989.}	43.9925	0.151864	newly 5 weeks cumul	27895.	33143.1	{21696., 54115.}	31.8224	0.115685	newly 56 weeks cumul	32739.	40066.1	{25421., 67169.}	35.5467	0.123991
5	newly 3 weeks spec	4803.	5189.62	{3102., 8280.}	29.9	0.122639	newly 3 weeks cumul	16297.	18955.1	{12532., 25668.}	28.0729	0.10868	newly 5 weeks spec	3233.	3268.87	{1786., 5340.}	37.9154	0.15989	newly 5 weeks cumul	23516.	26387.9	{16869., 40136.}	28.163	0.111779	newly 50 weeks cumul	34577.	36352.4	{21691., 56097.}	31.277	0.129214
6	newly 3 weeks spec	6383.	6720.07	{3743., 10358.}	34.8238	0.14457	newly 3 weeks cumul	23683.	26150.2	{17073., 37303.}	26.4334	0.106874	newly 5 weeks spec	2924.	3507.1	{1659., 5541.}	48.4408	0.169961	newly 5 weeks cumul	31005.	34586.4	{21284., 51109.}	30.4677	0.120757	newly 56 weeks cumul	36697.	41990.5	{24614., 62501.}	34.4011	0.131188
7	newly 3 weeks spec	5854.	8706.3	{5337., 12524.}	52.6233	0.16982	newly 3 weeks cumul	26186.	33313.9	{24433., 40996.}	30.5619	0.1131	newly 5 weeks spec	2014.	4364.77	{2349., 6239.}	117.395	0.291153	newly 5 weeks cumul	31770.	43988.5	{30385., 58449.}	41.4718	0.144231	newly 57 weeks cumul	34576.	52224.3	{34644., 70488.}	53.3437	0.173708
8	newly 3 weeks spec	4856.	7970.84	{5501., 10428.}	64.8145	0.205113	newly 3 weeks cumul	17949.	27014.7	{20007., 32569.}	52.8116	0.179969	newly 5 weeks spec	2677.	5449.66	{3048., 9469.}	104.171	0.27699	newly 5 weeks cumul	24358.	39176.	{28628., 49343.}	61.2079	0.1994	newly 53 weeks cumul	30079.	56068.8	{36957., 81733.}	86.6633	0.25481
9	newly 3 weeks spec	4469.	10454.6	{7349., 14692.}	133.937	0.355347	newly 3 weeks cumul	27073.	37787.5	{29463., 44013.}	41.2505	0.147882	newly 5 weeks spec	876.	5558.44	{3288., 8937.}	534.525	0.764084	newly 5 weeks cumul	30073.	51171.3	{40119., 62612.}	70.1569	0.224347	newly 58 weeks cumul	30570.	62154.7	{46155., 82363.}	103.319	0.297774
10	newly 3 weeks spec	5097.	7502.05	{5522., 9707.}	49.2427	0.166686	newly 3 weeks cumul	20668.	24586.6	{18086., 29583.}	27.0139	0.110577	newly 5 weeks spec	2025.	5572.89	{3524., 8114.}	175.204	0.415798	newly 5 weeks cumul	25993.	36701.2	{28219., 44955.}	42.8453	0.149681	newly 51 weeks cumul	29196.	58233.4	{40612., 81952.}	99.4566	0.284868
11	newly 3 weeks spec	4623.	7825.4	{5116., 12322.}	71.0769	0.211229	newly 3 weeks cumul	21770.	31250.3	{23027., 41912.}	45.433	0.15483	newly 5 weeks spec	1559.	3791.1	{2115., 6056.}	143.175	0.337365	newly 5 weeks cumul	26128.	40599.9	{28446., 57398.}	56.7922	0.182736	newly 57 weeks cumul	28027.	47792.1	{32351., 69953.}	71.1464	0.215041
12	newly 3 weeks spec	5357.	7227.12	{4740., 9277.}	40.1192	0.144773	newly 3 weeks cumul	18503.	23653.9	{17095., 27907.}	35.236	0.139592	newly 5 weeks spec	3340.	5375.68	{3553., 7361.}	63.0025	0.197312	newly 5 weeks cumul	26203.	35319.2	{25007., 43104.}	38.5149	0.13941	newly 50 weeks cumul	35015.	56897.	{40713., 76958.}	63.355	0.200742
13	newly 3 weeks spec	5099.	5429.75	{3091., 8615.}	32.5065	0.136398	newly 3 weeks cumul	17831.	19150.9	{12451., 27918.}	27.1068	0.113537	newly 5 weeks spec	3072.	3719.94	{1794., 6605.}	49.5246	0.177082	newly 5 weeks cumul	24961.	27402.2	{16588., 40899.}	30.5273	0.124522	newly 50 weeks cumul	33743.	40104.1	{21644., 65207.}	41.3571	0.152813
14	newly 3 weeks spec	4962.	6325.75	{3612., 9111.}	36.4338	0.132253	newly 3 weeks cumul	15685.	21140.3	{13966., 27120.}	42.4175	0.155457	newly 5 weeks spec	3640.	4675.49	{2495., 7712.}	44.3397	0.15602	newly 5 weeks cumul	23643.	31310.7	{19633., 42466.}	37.9229	0.135069	newly 50 weeks cumul	36706.	49442.2	{30008., 77112.}	43.2153	0.147081
15	newly 3 weeks spec	4360.	7502.05	{5522., 9707.}	73.0745	0.229022	newly 3 weeks cumul	17059.	24586.6	{18086., 29583.}	48.8077	0.174068	newly 5 weeks spec	2303.	5572.89	{3524., 8114.}	141.984	0.359929	newly 5 weeks cumul	22470.	36701.2	{28219., 44955.}	63.9973	0.208019	newly 52 weeks cumul	26744.	58233.4	{40612., 81952.}	117.744	0.322965
16	newly 3 weeks spec	5728.	7079.75	{5121., 9199.}	30.1461	0.11373	newly 3 weeks cumul	18259.	23670.4	{18680., 28398.}	33.403	0.127607	newly 5 weeks spec	4036.	5093.05	{3294., 7473.}	35.9445	0.126294	newly 5 weeks cumul	27272.	34838.3	{26267., 43394.}	31.4766	0.116488	newly 51 weeks cumul	38769.	54179.3	{37690., 73214.}	42.3485	0.1423
17	newly 3 weeks spec	4479.	9757.47	{6186., 13747.}	117.849	0.321015	newly 3 weeks cumul	20396.	34423.2	{23662., 41570.}	70.8158	0.226292	newly 5 weeks spec	1516.	5584.38	{2816., 9151.}	268.363	0.51587	newly 5 weeks cumul	24628.	47536.5	{34125., 59896.}	93.0182	0.276258	newly 57 weeks cumul	26798.	59572.9	{39504., 80642.}	122.304	0.331608
18	newly 3 weeks spec	4189.	8225.38	{5447., 11670.}	96.3566	0.276294	newly 3 weeks cumul	17056.	29464.1	{21345., 37190.}	72.7494	0.229445	newly 5 weeks spec	1958.	4934.84	{2636., 7729.}	152.035	0.355378	newly 5 weeks cumul	22027.	40905.6	{28915., 52764.}	85.7067	0.258478	newly 55 weeks cumul	25613.	53139.	{34753., 74098.}	107.469	0.299028
19	newly 3 weeks spec	3570.	6720.07	{3743., 10358.}	89.7947	0.24529	newly 3 weeks cumul	17052.	26150.2	{17073., 37303.}	55.6847	0.178744	newly 5 weeks spec	1298.	3507.1	{1659., 5541.}	170.192	0.36881	newly 5 weeks cumul	20704.	34586.4	{21284., 51109.}	68.8425	0.206892	newly 55 weeks cumul	22668.	41990.5	{24614., 62501.}	86.0692	0.240547
20	newly 3 weeks spec	4499.	7970.84	{5501., 10428.}	77.5072	0.236675	newly 3 weeks cumul	19401.	27014.7	{20007., 32569.}	42.4634	0.151088	newly 5 weeks spec	1849.	5449.66	{3048., 9469.}	194.735	0.434866	newly 5 weeks cumul	24272.	39176.	{28628., 49343.}	61.7675	0.200885	newly 53 weeks cumul	27031.	56068.8	{36957., 81733.}	107.424	0.300043
21	newly 3 weeks spec	5237.	6309.77	{3603., 9086.}	37.2274	0.131897	newly 3 weeks cumul	23703.	26257.8	{16958., 36883.}	25.4746	0.101946	newly 5 weeks spec	1742.	2912.12	{1543., 4259.}	72.6607	0.195884	newly 5 weeks cumul	28600.	33534.7	{20850., 47381.}	30.6006	0.114239	newly 57 weeks cumul	30632.	38858.4	{23566., 55161.}	37.0431	0.127824
22	newly 3 weeks spec	5006.	8202.83	{5074., 11358.}	65.9229	0.205915	newly 3 weeks cumul	21806.	28169.8	{19693., 34398.}	32.7311	0.119308	newly 5 weeks spec	1880.	5268.22	{2729., 9465.}	180.286	0.399695	newly 5 weeks cumul	26703.	40148.3	{27419., 51605.}	52.1285	0.17388	newly 55 weeks cumul	29097.	54187.1	{33743., 79890.}	86.7685	0.251882
23	newly 3 weeks spec	1987.	5774.55	{3214., 8363.}	190.617	0.41045	newly 3 weeks cumul	17807.	28418.2	{18085., 40819.}	62.4611	0.192106	newly 5 weeks spec	308.	1961.12	{988., 2840.}	536.726	0.729748	newly 5 weeks cumul	18940.	33810.4	{20778., 48560.}	80.361	0.230247	newly 59 weeks cumul	19129.	36274.	{21963., 52034.}	90.9849	0.251454
24	newly 3 weeks spec	4945.	7869.65	{4691., 12699.}	62.5767	0.182072	newly 3 weeks cumul	26504.	36710.	{26116., 49323.}	40.9715	0.140569	newly 5 weeks spec	1389.	2734.71	{1467., 4458.}	98.595	0.244797	newly 5 weeks cumul	30352.	44163.8	{30235., 64888.}	47.7428	0.156496	newly 60 weeks cumul	31658.	47622.3	{32131., 70220.}	52.3803	0.167326
25	newly 3 weeks spec	3439.	7869.65	{4691., 12699.}	128.835	0.32323	newly 3 weeks cumul	21402.	36710.	{26116., 49323.}	71.8433	0.223058	newly 5 weeks spec	698.	2734.71	{1467., 4458.}	291.792	0.535523	newly 5 weeks cumul	23735.	44163.8	{30235., 64888.}	86.1503	0.252913	newly 60 weeks cumul	24404.	47622.3	{32131., 70220.}	95.1415	0.271004
26	newly 3 weeks spec	5656.	8202.83	{5074., 11358.}	49.3723	0.164454	newly 3 weeks cumul	23609.	28169.8	{19693., 34398.}	25.2905	0.0969579	newly 5 weeks spec	2372.	5268.22	{2729., 9465.}	123.17	0.303561	newly 5 weeks cumul	29758.	40148.3	{27419., 51605.}	38.5692	0.136236	newly 54 weeks cumul	33191.	54187.1	{33743., 79890.}	65.2978	0.201887
27	newly 3 weeks spec	4510.	8378.47	{5412., 12039.}	86.0321	0.252735	newly 3 weeks cumul	21137.	29548.8	{22235., 36830.}	41.7772	0.146498	newly 5 weeks spec	1681.	5172.58	{2817., 8955.}	207.709	0.442862	newly 5 weeks cumul	25596.	41418.1	{28926., 52659.}	62.2847	0.200362	newly 55 weeks cumul	27946.	54681.1	{35271., 80637.}	95.7678	0.273292
28	newly 3 weeks spec	3624.	6119.93	{3200., 11198.}	74.2986	0.211214	newly 3 weeks cumul	14491.	23774.6	{14114., 35849.}	66.9151	0.204334	newly 5 weeks spec	2108.	3206.33	{1415., 6470.}	71.2713	0.205996	newly 5 weeks cumul	19406.	31487.1	{18132., 51775.}	65.8825	0.198444	newly 54 weeks cumul	24332.	38231.9	{20687., 64866.}	63.538	0.189489
29	newly 3 weeks spec	4912.	7745.56	{4988., 11436.}	60.6981	0.186996	newly 3 weeks cumul	20248.	28644.6	{21247., 36494.}	43.686	0.149996	newly 5 weeks spec	2132.	4314.21	{2357., 6787.}	104.434	0.26397	newly 5 weeks cumul	25828.	38847.5	{27067., 52750.}	52.7235	0.171947	newly 55 weeks cumul	29513.	48584.2	{32066., 68328.}	66.5693	0.202565
30	newly 3 weeks spec	4348.	7320.48	{4951., 10010.}	72.0514	0.228252	newly 3 weeks cumul	16097.	23823.8	{18402., 28970.}	56.2906	0.205581	newly 5 weeks spec	2202.	5429.75	{3091., 8615.}	146.582	0.362752	newly 5 weeks cumul	21394.	35654.2	{25743., 45283.}	69.1517	0.222222	newly 51 weeks cumul	26261.	56607.4	{36554., 80321.}	115.557	0.315107
31	newly 3 weeks spec	1715.	6110.66	{2955., 10415.}	256.307	0.493391	newly 3 weeks cumul	14771.	31339.6	{17807., 49416.}	112.365	0.299798	newly 5 weeks spec	342.	1878.4	{843., 3258.}	449.239	0.661249	newly 5 weeks cumul	15873.	36682.5	{20279., 58569.}	131.164	0.331361	newly 60 weeks cumul	16071.	38831.4	{21228., 62369.}	141.624	0.348285
32	newly 3 weeks spec	4733.	7715.65	{4417., 11039.}	65.8097	0.194105	newly 3 weeks cumul	23783.	32761.9	{21819., 42623.}	40.5273	0.14008	newly 5 weeks spec	1187.	3203.87	{1636., 4566.}	169.913	0.378771	newly 5 weeks cumul	27483.	41048.7	{26159., 55366.}	51.337	0.166667	newly 58 weeks cumul	28771.	45972.5	{28531., 62294.}	60.9058	0.188771
33	newly 3 weeks spec	3999.	6358.2	{3607., 11159.}	64.4936	0.182825	newly 3 weeks cumul	20824.	28662.8	{18780., 45181.}	43.0684	0.142391	newly 5 weeks spec	1142.	2511.43	{1273., 4569.}	121.169	0.285615	newly 5 weeks cumul	24133.	35232.	{22301., 56843.}	50.835	0.1598	newly 58 weeks cumul	25686.	39033.	{24193., 63798.}	56.2285	0.171245
34	newly 3 weeks spec	4878.	7227.12	{4740., 9277.}	51.777	0.176763	newly 3 weeks cumul	19185.	23653.9	{17095., 27907.}	31.3765	0.128065	newly 5 weeks spec	2525.	5375.68	{3553., 7361.}	112.898	0.309047	newly 5 weeks cumul	25181.	35319.2	{25007., 43104.}	43.3249	0.153232	newly 50 weeks cumul	30277.	56897.	{40713., 76958.}	87.9216	0.259964
35	newly 3 weeks spec	3516.	9455.74	{5978., 15377.}	168.935	0.400579	newly 3 weeks cumul	21757.	37942.9	{28639., 48944.}	74.3939	0.232551	newly 5 weeks spec	674.	4057.72	{2110., 7093.}	502.036	0.72341	newly 5 weeks cumul	24081.	48358.8	{34569., 67178.}	100.817	0.289169	newly 59 weeks cumul	24720.	54671.3	{37734., 79946.}	121.162	0.327366
36	newly 3 weeks spec	6059.	9586.95	{6589., 13558.}	58.9592	0.188582	newly 3 weeks cumul	27874.	34879.1	{27586., 40998.}	28.0782	0.106225	newly 5 weeks spec	2044.	5277.76	{3127., 8770.}	158.207	0.37441	newly 5 weeks cumul	33550.	47455.1	{35514., 60234.}	42.1681	0.14671	newly 57 weeks cumul	35952.	58756.2	{41516., 81080.}	63.8867	0.203522
37	newly 3 weeks spec	2491.	7782.8	{4769., 11684.}	212.437	0.460982	newly 3 weeks cumul	16769.	34465.	{24794., 45973.}	105.528	0.300111	newly 5 weeks spec	544.	3005.05	{1694., 4679.}	452.399	0.688919	newly 5 weeks cumul	18580.	42415.7	{29219., 59939.}	128.287	0.34113	newly 59 weeks cumul	19084.	46723.2	{31460., 66488.}	144.829	0.368704
38	newly 3 weeks spec	4614.	7825.4	{5116., 12322.}	71.3729	0.211911	newly 3 weeks cumul	23415.	31250.3	{23027., 41912.}	36.6552	0.129665	newly 5 weeks spec	1497.	3791.1	{2115., 6056.}	153.246	0.354989	newly 5 weeks cumul	27574.	40599.9	{28446., 57398.}	49.5849	0.16387	newly 57 weeks cumul	29436.	47792.1	{32351., 69953.}	63.6515	0.196837
39	newly 3 weeks spec	3711.	5420.54	{2560., 9520.}	59.607	0.188929	newly 3 weeks cumul	14691.	21343.9	{11544., 32145.}	53.5971	0.175411	newly 5 weeks spec	1875.	2913.69	{1153., 5384.}	74.946	0.213167	newly 5 weeks cumul	19116.	28309.	{14308., 46264.}	57.6562	0.184646	newly 54 weeks cumul	22836.	34820.	{16488., 58783.}	63.2423	0.193589
40	newly 3 weeks spec	4019.	7344.2	{3656., 13349.}	89.1936	0.245256	newly 3 weeks cumul	16800.	29831.7	{17053., 42876.}	81.5348	0.243247	newly 5 weeks spec	1644.	3268.89	{1215., 6384.}	110.362	0.267493	newly 5 weeks cumul	20945.	38099.9	{22093., 59112.}	85.3696	0.247042	newly 57 weeks cumul	23590.	43422.4	{23881., 68776.}	88.1324	0.248945
41	newly 3 weeks spec	3584.	5701.44	{2939., 9760.}	66.5316	0.184781	newly 3 weeks cumul	19699.	28049.9	{16749., 45172.}	48.3547	0.157231	newly 5 weeks spec	987.	1935.97	{885., 3289.}	100.608	0.237841	newly 5 weeks cumul	22587.	33375.7	{19324., 54375.}	53.5562	0.167021	newly 59 weeks cumul	23519.	35814.4	{20422., 58483.}	57.646	0.175209
42	newly 3 weeks spec	4951.	7976.1	{5517., 10469.}	62.7693	0.204144	newly 3 weeks cumul	19337.	25764.4	{19927., 30611.}	38.8606	0.147598	newly 5 weeks spec	2604.	5928.95	{3499., 9374.}	127.686	0.331	newly 5 weeks cumul	25605.	38667.2	{28675., 47645.}	52.2873	0.177558	newly 52 weeks cumul	30663.	60626.1	{39949., 84814.}	97.7175	0.281371
43	newly 3 weeks spec	3668.	7320.48	{4951., 10010.}	102.079	0.294695	newly 3 weeks cumul	13660.	23823.8	{18402., 28970.}	81.3417	0.265559	newly 5 weeks spec	2047.	5429.75	{3091., 8615.}	165.254	0.394451	newly 5 weeks cumul	18398.	35654.2	{25743., 45283.}	95.3874	0.282531	newly 51 weeks cumul	23176.	56607.4	{36554., 80321.}	144.25	0.36938
44	newly 3 weeks spec	5502.	9836.76	{6696., 14768.}	78.7851	0.234558	newly 3 weeks cumul	26971.	36299.2	{28646., 44392.}	36.1461	0.13069	newly 5 weeks spec	1807.	5169.92	{2804., 8757.}	186.105	0.404388	newly 5 weeks cumul	31936.	48807.1	{36803., 62825.}	52.9161	0.176456	newly 58 weeks cumul	33949.	58704.1	{41861., 81783.}	72.9184	0.224458
45	newly 3 weeks spec	5745.	8733.67	{5501., 12891.}	55.4977	0.177072	newly 3 weeks cumul	23088.	31557.3	{22268., 39733.}	39.1658	0.138244	newly 5 weeks spec	2549.	4929.11	{2527., 7571.}	95.5752	0.245382	newly 5 weeks cumul	29573.	43206.5	{30079., 57374.}	47.9665	0.160965	newly 56 weeks cumul	33764.	54117.6	{35093., 75750.}	61.7244	0.192336
46	newly 3 weeks spec	5945.	8804.21	{6090., 11525.}	50.3728	0.169371	newly 3 weeks cumul	25387.	30261.6	{23630., 36112.}	25.4946	0.104221	newly 5 weeks spec	2372.	5688.23	{3225., 9960.}	139.807	0.346419	newly 5 weeks cumul	31718.	43162.5	{31963., 53003.}	37.8702	0.134858	newly 55 weeks cumul	35057.	59076.2	{40175., 83849.}	68.565	0.213714
47	newly 3 weeks spec	4772.	6693.69	{4541., 8801.}	45.3705	0.155917	newly 3 weeks cumul	17003.	23340.5	{16339., 29193.}	41.0501	0.145106	newly 5 weeks spec	2927.	4487.16	{2518., 6728.}	60.9564	0.18427	newly 5 weeks cumul	23808.	33371.4	{23625., 42886.}	44.1685	0.153762	newly 51 weeks cumul	31769.	47426.8	{30498., 66590.}	53.9406	0.174138
48	newly 3 weeks spec	5735.	7983.66	{5031., 12455.}	46.3788	0.154089	newly 3 weeks cumul	21931.	30449.	{22402., 37672.}	40.8267	0.141982	newly 5 weeks spec	2847.	4122.16	{2172., 6858.}	57.8742	0.174131	newly 5 weeks cumul	29108.	40415.3	{28033., 54789.}	42.2873	0.144412	newly 56 weeks cumul	34186.	48600.4	{31978., 70657.}	46.4836	0.153226
49	newly 3 weeks spec	3521.	7869.65	{4691., 12699.}	123.506	0.312996	newly 3 weeks cumul	23225.	36710.	{26116., 49323.}	58.8801	0.189929	newly 5 weeks spec	685.	2734.71	{1467., 4458.}	299.228	0.543688	newly 5 weeks cumul	25469.	44163.8	{30235., 64888.}	73.9159	0.224266	newly 60 weeks cumul	25996.	47622.3	{32131., 70220.}	83.4731	0.244812
50	newly 3 weeks spec	2974.	5774.55	{3214., 8363.}	96.7547	0.24812	newly 3 weeks cumul	18646.	28418.2	{18085., 40819.}	56.0012	0.175886	newly 5 weeks spec	685.	1961.12	{988., 2840.}	186.436	0.383229	newly 5 weeks cumul	20804.	33810.4	{20778., 48560.}	65.6369	0.196002	newly 59 weeks cumul	21381.	36274.	{21963., 52034.}	72.375	0.209997
